# Biodegradable, Flexible and Ultraviolet Blocking Nanocellulose Composite Film Incorporated with Lignin Nanoparticles

**DOI:** 10.3390/ijms232314863

**Published:** 2022-11-28

**Authors:** Huiyang Bian, Xuan Shu, Wenhao Su, Dan Luo, Maolin Dong, Xiuyu Liu, Xingxiang Ji, Hongqi Dai

**Affiliations:** 1 State Key Laboratory of Biobased Material and Green Papermaking, Qilu University of Technology, Shandong Academy of Sciences, Jinan 250353, China; 2 Jiangsu Co-Innovation Center of Efficient Processing and Utilization of Forest Resources, International Innovation Center for Forest Chemicals and Materials, Nanjing Forestry University, Nanjing 210037, China; 3 Key Laboratory of Chemistry and Engineering of Forest Products, State Ethnic Affairs Commission, Guangxi Key Laboratory of Chemistry and Engineering of Forest Products, Guangxi Minzu University, Nanning 530006, China

**Keywords:** nanocellulose, lignin nanoparticles, composite film, ultraviolet blocking, biodegradation

## Abstract

The exploration of functional films using sustainable cellulose-based materials to replace plastics has been of much interest. In this work, two kinds of lignin nanoparticles (LNPs) were mixed with cellulose nanofibrils (CNFs) for the fabrication of composite films with biodegradable, flexible and ultraviolet blocking performances. LNPs isolated from *p*-toluenesulfonic acid hydrolysis was easily recondensed and deposited on the surface of composite film, resulting in a more uneven surface; however, the composite film consisting of CNFs and LNPs isolated from maleic acid hydrolysis exhibited a homogeneous surface. Compared to pure CNF film, the composite CNF/LNP films exhibited higher physical properties (tensile strength of 164 MPa and Young’s modulus of 8.0 GPa), a higher maximal weight loss temperature of 310 °C, and a perfect UVB blocking performance of 95.2%. Meanwhile, the composite film had a lower environmental impact as it could be rapidly biodegraded in soil and manmade seawater. Overall, our results open new avenues for the utilization of lignin nanoparticles in biopolymer composites to produce functional and biodegradable film as a promising alternative to petrochemical plastics.

## 1. Introduction

Due to the industrial emissions induced by human activities, the ozone layer has become thinner, exposing the surface of the Earth to increasing ultraviolet radiation [1,2]. According to different wavelengths of radiation, ultraviolet light was divided into three bands, which were UVA (320~400 nm), UVB (280~320 nm), and UVC (190~280 nm) [3]. Among them, UVA and UVB bands had strong ultraviolet penetration, which not only posed a threat to human health, but also affected the growth of animals and plants, and accelerated the aging and degradation of daily materials [4,5]. Until now, commercial UV shielding films were mostly composed of petroleum-based polymers such as polyethylene (PE), but these polymers were naturally difficult to degrade, and could form microplastics with smaller particle size through the actions of light, heat, impact, and wear, which endangered the ecological environment and human life and health [6]. Therefore, it is vital to develop environmentally friendly and excellent UV shielding film materials using non petroleum-based materials.

As the world’s most abundant resource, cellulose is an alternative to petroleum-based polymers because of its outstanding biodegradability and biocompatibility [7,8]. Among various cellulosic materials, cellulose nanofibrils (CNFs) have attracted considerable attention because of their high mechanical strength, high transparency, barrier property, etc. [9,10]. It has been found that the introduction of ultraviolet shielding agents into CNFs could prepare ultraviolet shielding film materials. The commonly used ultraviolet shielding agents included organic photoactive materials (benzophenone, diisocyanate, etc.) and inorganic oxides (titanium dioxide, zinc oxide, etc.) [11,12,13]. However, these UV shielding agents had several problems, such as poor light stability, easy degradation, and re-aggregation, which limit their development potential in UV shielding film materials [14].

Lignin is one of the three major components in the plant cell wall, which contains functional groups, such as aryl, phenolic hydroxyl, and ketones, making it suitable for use as a UV shielding agent [15,16]. So far, there have been some reports that utilizing lignin can improve the UV shielding performance of nanocellulose films. For example, Sadeghifar et al. prepared cellulose nanocrystals/lignin composite film materials by reacting azide-modified cellulose nanocrystals with propargylic lignin to form covalent bonds through click chemistry. The UVA and UVB shielding efficiency of the composite film reached 90% and 100%, respectively, when the lignin content was 2%. However, this method was not suitable for industrial application because of its harsh reaction conditions and complex preparation process [17]. In our previous work, natural lignocellulosic nanofibrils (LCNFs) consisting of lignin and CNFs was successfully produced from poplar chemimechanical pulp. LCNF films with various lignin contents (4.89–15.68%) were prepared and corresponding UVA and UVB blocking rates were as high as 99.5% and 100%, respectively. However, this method could still not achieve accurate adjustment of lignin content in the composite films [18]. Farooq et al. added certain amounts of colloidal kraft lignin particles into CNFs to prepare composite film by filtration and drying processes. This method could regulate the proportion of lignin in the composite film and realize excellent UV shielding performance. However, the transmittance at 600 nm was less than 40% when the lignin content was increased to 12%, probably due to not reducing the size of lignin [19]. Therefore, it is necessary to consider the influence of lignin size and dispersibility on the physiochemical properties of CNF/lignin composite film.

Herein, two kinds of lignin nanoparticles (LNPs) were isolated from poplar sawdust using maleic acid (MA) and *p*-toluenesulfonic acid hydrolysis (PA). The size of the LNPs was tuned by controlling the acid treatment conditions. Then, LNPs with different contents were mixed with CNFs to prepare composite film through filtration and pressing processes. The morphology and diameter of different LNPs were first compared. Then, the morphology, physical property, thermal stability, UV shielding performance, surface chromaticity value, and biodegradation of composite films were discussed. This work is aimed at not only achieving the quantification of LNPs and CNFs in composite film, but to also elucidate the relationship between LNP diameter and film properties, which can open up broader prospects for lignin nanoparticles in food packing material, UV protection, and surface coating for vehicles.

## 2. Results

### 2.1. Characterization of LNPs

**Figure 1:**
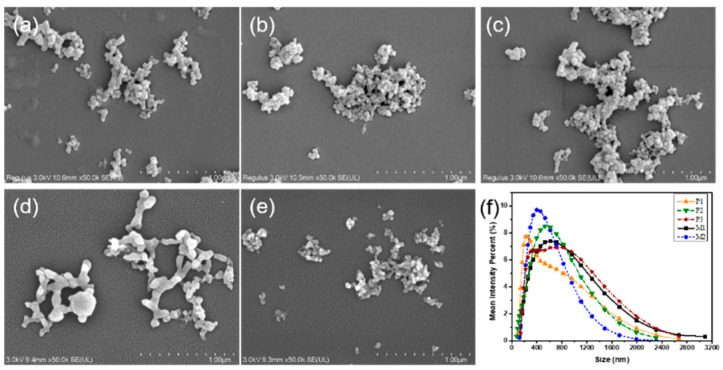
FE-SEM images of different lignin isolated from *p*-toluenesulfonic acid and maleic acid hydrolysis under different reaction conditions. (**a**) P1; (**b**) P2; (**c**) P3; (**d**) M1; (**e**) M2. Magnification: 50,000×. (**f**) Mean intensity percent of different LNP size.

LNPs isolated from *p*-toluenesulfonic acid and maleic acid hydrolysis were observed to investigate their morphology using FE-SEM images (Figure 1a–f). LNP size was tuned by adjusting the acid treatment conditions (Table S1). For simplicity, LNP specimens were abbreviated as P1, P2, P3, M1, and M2. The results verified that LNPs were formed through the self-assembly behavior of dissolved lignin after precipitation [20]. Some spherical LNPs were aggregated during the drying process, having influence on the actual measured size of the LNPs. With the increased hydrolysis severity, the size of PA-isolated LNPs increased; however, MA-isolated LNPs exhibited the opposite trend. The specific size of different LNPs was detected by dynamic light scattering, as shown in Figure 1g–h. The DLS size of PA-isolated LNPs increased substantially from 341 to 460 nm when the hydrolysis severity was enhanced; this suggested that lignin condensation may have occurred under strong acid treatment conditions [21,22]. On the contrary, the DLS size of MA-isolated LNPs was decreased from 533 to 410 nm with increasing hydrolysis duration, perhaps due to the weaker acidity of MA, which only broke the chemical bonds (such as ether and ester bonds) between lignin and carbohydrates without causing lignin condensation.

### 2.2. Surface Morphology of Composite Film

**Figure 2:**
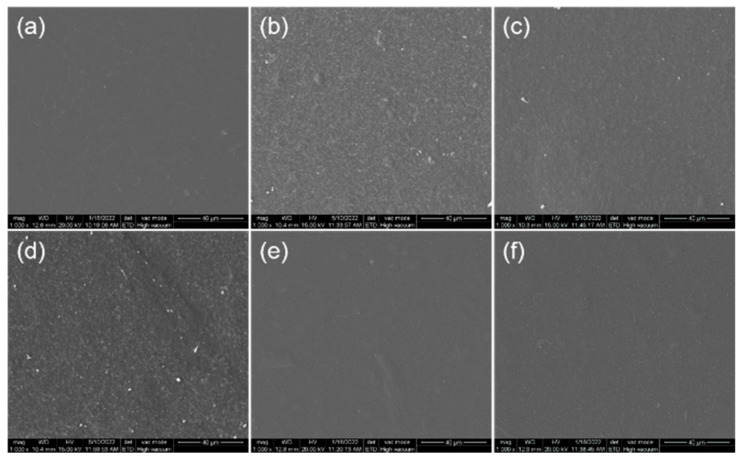
SEM images of pure CNFs and different composite films. (**a**) CNF; (**b**) CNF/P1; (**c**) CNF/P2; (**d**) CNF/P3; (**e**) CNF/M1; (**f**) CNF/M2. Magnification: 1000×.

SEM images were used to observe the assembly behavior of CNFs and different kinds of LNPs during composite film formation (Figure 2). It can be seen that the pure CNF film exhibited a uniform and smooth surface due to the strong bonding between nanofibrils. When two kinds of LNPs were added into the CNF suspension, respectively, the resulting composite film became uneven with a small number of irregular particles attached. In addition, when the PA-isolated LNPs increased from 341 to 460 nm, the corresponding composite film became rougher with comparatively coarse aggregation. Compared to PA-isolated LNPs, it seemed that the composite film consisted of CNFs and LNPs isolated from MA hydrolysis exhibited a homogeneous surface. This may be ascribed to the following reasons: (1) LNP isolated from PA hydrolysis was easily recondensed and deposited on the film, resulting in an uneven composite film surface; (2) LNP isolated from MA hydrolysis was esterified with MA, which improved the surface charge of the LNPs and electrostatic repulsive interaction with CNF [23]. Thus, the existence of LNPs made the composite film comparatively unsmooth, and the size and surface charge of the LNPs were the main influencing factors.

### 2.3. Physical and Tensile Property

**Figure 3:**
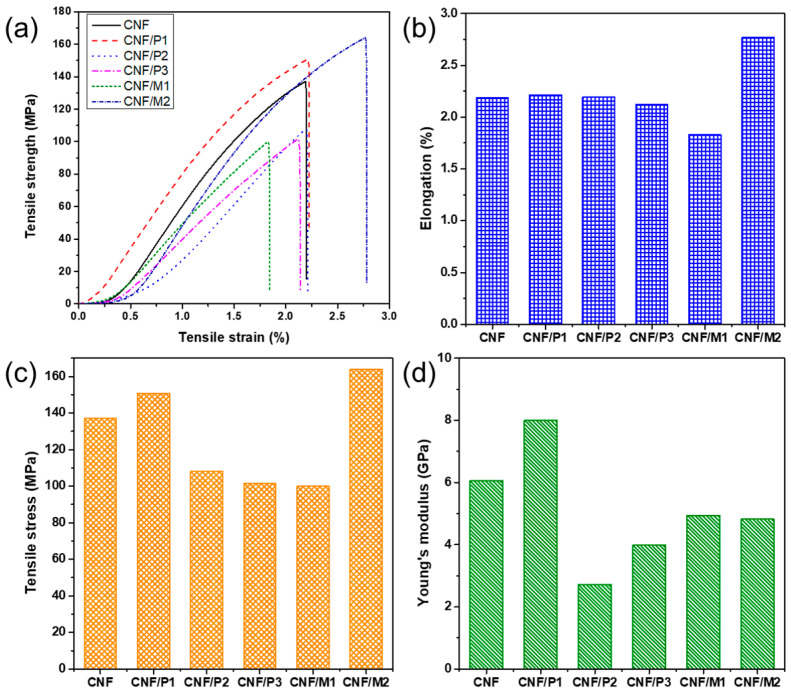
Mechanical properties of pure CNF film and different composite films. (**a**) Stress–strain curve; (**b**) Elongation to break; (**c**) Tensile stress; (**d**) Young’s modulus.

The presence of LNPs in composite film affected the mechanical properties, such as tensile stress, elongation, and Young’s modulus. Figure 3a showed the stress–strain curves of pure CNF films and composite films with various LNP samples. The tensile properties of the composite film are related to the single fibril, the bonding force between fibrils, and actual processing conditions [24]. Compared to composite film, pure CNF film had an elongation to break of 2.2%, a tensile strength of 137.0 MPa, and a Young’s modulus of 6.1 GPa. After mixing with PA-isolated LNPs, the elongation was kept almost constant, but the tensile stress was reduced with the decreasing size of LNPs, which was due to the lignin aggregation that hindered the formation of hydrogen bonding. Similar to PA-isolated LNPs, adding MA-isolated LNPs also resulted in decreased tensile stress; however, increasing MA hydrolysis duration endowed LNPs with more carboxyl groups where LNPs could act as a cross-linker between chains in the composite film, resulting in a moderate increase in the tensile strength of the composite film (164 MPa) relative to the pure film. Overall, these results obtained here were far higher than our work previously reported for LCNF film (82.7 MPa) produced from chemomechanical pulp fibers [18]. Another property, Young’s modulus, was calculated from the initial linear region (approximately 1.0%) of the stress–strain curve. The CNF/P1 film exhibited the highest Young’s modulus of 8.0 GPa among all the composite films. One possible reason for this was that the size of P1 was very small and could be finely embed within CNFs without destroying the intermolecular hydrogen bonding [25]. At this point, improving the physical properties of composite films can be achieved by tailoring the size or surface of the functional groups of lignin.

### 2.4. Thermal Stability

**Figure 4:**
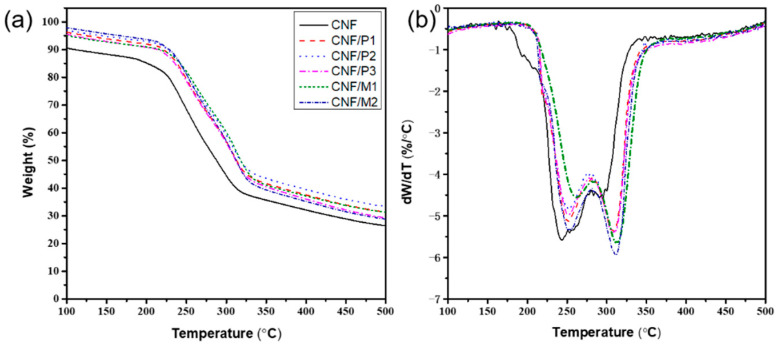
(**a**) TG and (**b**) DTG curves of pure CNF film and different composite films.

The thermal degradation performance of composite film is regarded as an important parameter in packaging, insulating, and gas separation materials [26]. The weight loss and derivative thermogravimetry curves of all prepared films were measured via TGA and DTG in the temperature range from 100–500 °C, as shown in Figure 4. It was clear that pure CNF film decomposed relatively easily at the beginning of the heating stage due to the moisture absorbed on the surface of CNF film. With the addition of LNPs, the CNF/LNP composite film displayed better thermal stability with higher maximal weight loss temperature (T_max_) (Figure 4b). Different from reported CNF/lignin composite films [19], DTG curves had two apparent peaks, where the first one at a lower temperature was ascribed to the carboxyl groups of CNFs generated by the use of TEMPO oxidation technology [27], and the second one at a higher temperature originated from the presence of lignin containing various aromatic groups, ether and carbon–carbon bonds [28]. Compared with pure CNF film (T_max_ of 243 °C), all CNF/LNP composite films possessed higher T_max_ of approximately 310 °C, broadening their applications in thermal insulating and packaging materials.

### 2.5. UV Blocking Performance

**Figure 5:**
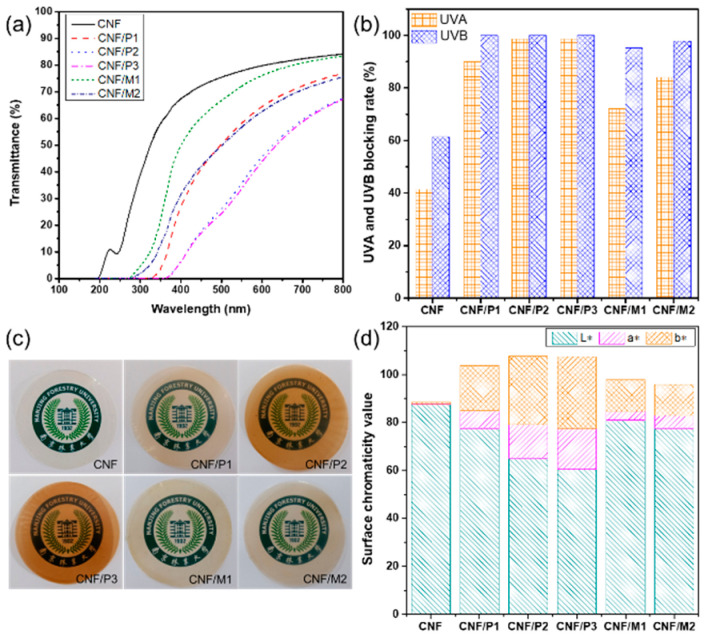
(**a**) The UV-vis transmission spectra of pure CNF film and different composite films. (**b**) UVA and UVB blocking rate of pure CNF film and different composite films. (**c**) Visual photograph of pure CNF film and different composite films. (**d**) Surface chromaticity value of pure CNF film and different composite films.

High transparency and strong UV absorption is important for films used in the packaging of food and medicine fields [29]. The presence of phenolic rings in LNPs are known to exhibit UV blocking properties [30]. To analyze the UV blocking performance of the composite films, UV-vis transmission spectra of pure CNF film and different composite films are compared in Figure 5a. For pure CNF film, UV light could not be absorbed and the transmittance value of the pure CNF film was 79.8% at a wavelength of 600 nm. Incorporating lignin into the composite film led to a significant decrease in light transmittance value, especially when mixing PA-isolated LNPs. Comparing LNPs obtained by PA and MA, CNF/M1 displayed a relatively higher transmittance value of 76.1%. The main reason was attributed to the lighter color of M1 in all LNP samples. Nonetheless, CNF/M1 also showed good UV blocking performance with 72.0 and 95.2% shielding capabilities for UVA and UVB, respectively. Huang et al. added nanoscale lignin from prehydrolysate into CNF suspension to prepare the lignin-reinforced CNF composite film, showing the same results with our work in that the composite film exhibited excellent UV protection capabilities [25]. This remarkable UV blocking performance was attributed to the UV-absorbing functional groups in lignin, including phenolic units, ketones, and other chromophores, making it a natural broad-spectrum blocker.

Figure 5c intuitively shows the photographs of pure CNF film and different composite films, where the color was changed from transparent to brownish yellow due to the different components in the films. Surface chromaticity value was measured to quantify the color of the composite film, shown in Figure 5d. Among all the films, the pure CNF film exhibited the highest L value (87.5) and the lowest a and b value (0.2 and 0.8), suggesting that this sample was relatively transparent. After mixing with LNPs, the L value was decreased; however, the a and b values of the composite film were increased. In particular, the a and b values rose more significantly, from 0.2 to 16.9 and 0.8 to 29.9, respectively. These results indicated that the red and yellow colors of the composite films became darker and UV was easily absorbed in these visible parts of the spectrum. In addition, compared to MA-isolated LNPs, CNF/P1-P3 showed higher a and b values, proving better UV blocking performance of the composite films.

### 2.6. Environment Impacts of Composite Film

**Figure 6:**
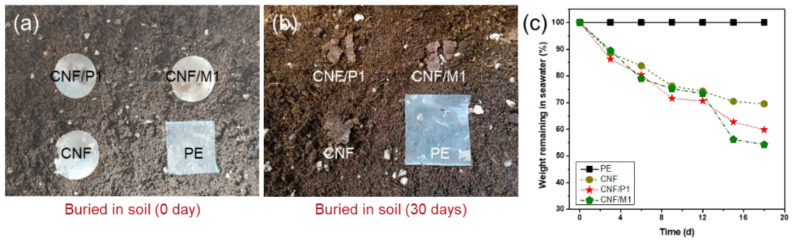
(**a**,**b**) The biodegradability test under moist soil for pure CNF, CNF/P1, CNF/M1, and PE films. (**c**) Effect of seawater immersion time on the weight remaining of pure CNF, CNF/P1, CNF/M1, and PE films.

The composite film exhibited excellent biodegradability in the natural environment. For example, three composite films (CNF/P1, CNF/M1, and CNF) and polyethylene (PE) were buried in soil at the same depth to monitor their morphology over time. After one month of burial in the soil, three composite films became fractured, likely due to the existence of microorganisms, which could directly digest and degrade the cellulose and lignin macromolecules in composite films [31]. This result was consistent with the work reported by Dong et al. in that paper straw made from pine was fully biodegradable after 30 days [32]. In comparison, PE film maintained original shape without any reduction in mass after being buried for the same amount of time, suggesting this non-biodegradable plastic posed a long-lasting burden on the environment (Figure 6a,b). Meanwhile, the mass changes in the composite films and PE in sea water were also evaluated (Figure 6c). After 18 days, the mass of CNF, CNF/P1, and CNF/M1 films were reduced by approximately 30%, 40%, and 45%, respectively. The mass loss of composite film was mainly due to the existence of microorganisms and ions in sea water. Meanwhile, compared with pure CNF film, CNF/LNP film showed better biodegradability, possibly due to the presence of more hydrogen bonds between fibrils in the pure CNF film that could not be easily broken. Similar to being buried in soil, PE film also remained stable in sea water without any change in mass. These results suggested that pure CNF film and CNF/LNP composite films were biodegradable in the nature and environmentally benign.

## 3. Materials and Methods

### 3.1. Materials

Cellulose nanofibrils (CNFs) were purchased from ScienceK Ltd, Zhejiang, China. Woody poplar sawdust (PS) was harvested from Jiangsu, China, and stored at room temperature. Maleic acid and *p*-toluenesulfonic acid were purchased from Aladdin Chemical Co. Ltd. (Shanghai, China).

### 3.2. Isolation of Different LNPs

LNPs were isolated from PS using MA and PA hydrolysis as previously described [21]. In brief, PS of 10 g was added into the fully dissolved acid solution to ensure a solid to liquor mass ratio of 1:10. Reaction was conducted at desired acid concentration, hydrolysis temperature, and duration, and was terminated by adding 100 mL of DI water. The filtrate was collected and diluted using DI water. LNPs were successfully isolated through centrifuging diluted liquor at 8000 rpm several times until the supernatant was turbid.

### 3.3. Composite Film Preparation

The CNF suspension was diluted to 0.5 wt% consistency and stirred for 1 h for pure a CNF film or mixed with different LNP suspensions for a CNF/LNP composite film. LNP content was 5 wt% on the basis of total dry weight in the composite film. The mixture was stirred for 1 h to achieve a well-uniformed suspension, then composite films with different types and contents of LNPs were prepared using vacuum filtration. The formed CNF/LNP film was placed between two commercial filter papers (with a diameter of 9 cm) and copper plates, then they were pressed for 48 h at 25 °C and 60 °C, respectively, under a weight of 5 kg.

### 3.4. Characterizations

#### 3.4.1. Particle Size Determination

A dynamic light scattering analyzer (DLS, Malvern Instruments Ltd., Zetasizer Nano Series Model Nano ZS, Malvern, UK) was used to measure the effective diameters of LNPs. Each sample with a concentration of approximately 1 g/L was measured and the results were presented as mean values.

#### 3.4.2. Microstructure Analysis

The morphology of LNPs was characterized using a cold filed-emission scanning electron microscope (CF-SEM, Regulus 8100, Hitachi, Hitachi, Japan). The surface microstructure of the CNF/LNP composite film was measured using scanning electron microscopy (SEM, Quanta 200, FEI, Columbus, OH, USA). Samples were fixed onto mounts, then coated with gold before testing.

#### 3.4.3. Mechanical Property of Composite Film

The TRAPPEZIUM X type tester (Shimadzu Corporation, Tokyo, Japan) was used to measure the mechanical properties (tensile stress and Young’s modulus) of the composite films. The load cell and crosshead speed was set to 500 N and 1 mm/min, respectively. Each film was tested for five measurements and the average value was reported.

#### 3.4.4. Thermogravimetric Analysis (TGA)

The thermal stability of composite films was evaluated by thermogravimetric analyzer (NETZSCH, TG 209F1, Selb, Germany) with a heating temperature from 50–600 °C (10 °C/min in increment) under nitrogen protection of 20 mL/min [33].

#### 3.4.5. UV-Vis Spectroscopy

The UV-Vis transmittance was measured using a spectrophotometer (UV-1780, Shimadzu, Japan) with a wavelength from 200–900 nm. The UVA and UVB blocking rates of the composite films were calculated by Equations (1) and (2):

(1) $$\mathrm{UVA}\;\mathrm{blocking}=1-\frac{\int_{320}^{400}T_{\lambda }\times d\lambda }{\int_{320}^{400}d\lambda }\;$$

(2) $$\mathrm{UVB}\;\mathrm{blocking}=1-\frac{\int_{280}^{320}T_{\lambda }\times d\lambda }{\int_{280}^{320}d\lambda }\;$$

where T_λ_ is the average spectral transmittance, d(λ) is the bandwidth, and λ is the wavelength.

#### 3.4.6. Surface Chromaticity Value

The surface chromaticity value of the composite films was analyzed using an Xrite i1 Pro2 spectrophotometer. CIELAB consisted of three components related to color (L, a, and b) and was specified by the International Commission on Illumination (CIE). Specifically, L represents the lightness of the color in which low numbers (0–50) and high numbers (51–100) refer to darkness and lightness, respectively. The values of a and b range from +127 to −128, where a includes green (−a) and red (+a), and b includes yellow (+b) and blue (−b).

#### 3.4.7. Biodegradation Testing

The biodegradation test was carried out in soil and seawater, which simulates biodegradability under real-life conditions. A standard composition of seawater included 26.7 g/L NaCl, 0.7 g/L KCl, 2.3 g/L MgCl_2_, and 1.2 g/L CaCl_2_. In the biodegradation process, the composite films and polyethylene (PE) were buried at the same depth in the soil at ambient temperature or immersed in prepared sea water solution. The degradation performance of the composite films in soil and sea water were observed after one month and 18 days, respectively.

## 4. Conclusions

In summary, CNF composite films were fabricated using facile vacuum filtration and the pressing process by incorporating different types of LNPs. Although the addition of LNPs resulted in a composite film with an uneven surface and decreased mechanical property, reducing the size of the LNPs or increasing the maleic acid reaction duration could improve the tensile strength and Young’s modulus to 164 MPa and 8 GPa, respectively, compared to pure CNF film. Apart from strength enhancement, the composite film also showed excellent UV resistance and thermal stability. Compared to petrochemical-based plastics such as PE, the composite film was biodegradable in nature and friendly to the environment. Thus, this work provided a facile strategy to prepare biodegradable and multifunctional film from natural lignocellulosic biopolymers to replace conventional plastics towards sustainable fields, such as agricultural mulching films and packaging materials.

## Data Availability

All data generated or analyzed during this study are included in this published article and its additional files.

## Supplementary Materials

The following supporting information can be downloaded at https://www.mdpi.com/article/10.3390/ijms232314863/s1, Table S1: List of *p*-toluenesulfonic acid and maleic acid hydrolysis experiment conditions of woody poplar sawdust.
