# Supplementary material for: Biodegradable, Flexible and Ultraviolet Blocking Nanocellulose Composite Film Incorporated with Lignin Nanoparticles
Source: Int J Mol Sci. 2022 Nov 28;23(23):14863. doi: 10.3390/ijms232314863 (PMC9735624; doi:10.3390/ijms232314863)
Supplement: Supplementary file 1 [file ijms-23-14863-s001.zip › ijms-1998308-supplementary.pdf]

Table S1 List of *p*-toluenesulfonic acid and maleic acid hydrolysis experiment conditions of woody poplar sawdust.

LNP sample label	Acid concentration (wt%)	Temperature (°C)	Hydrolysis (min)
P1	40	80	60
P2	60	80	120
P3	80	80	20
M1	50	100	60
M2	50	100	120
